# Platform for Real-Time Simulation of Dynamic Systems and Hardware-in-the-Loop for Control Algorithms

**DOI:** 10.3390/s141019176

**Published:** 2014-10-15

**Authors:** Isaac D. T. de Souza, Sergio N. Silva, Rafael M. Teles, Marcelo A. C. Fernandes

**Affiliations:** Department of Computer Engineering and Automation, Center of Technology, Federal University of Rio Grande do Norte—UFRN, Natal 59078-970, Brazil; E-Mails: isaacdiego@gmail.com (I.D.T.S.); s.natansilva@gmail.com(S.N.); rmteles@gmail.com (R.T.)

**Keywords:** real time, simulation, dynamic systems, embedded systems, hardware-in-the-loop

## Abstract

The development of new embedded algorithms for automation and control of industrial equipment usually requires the use of real-time testing. However, the equipment required is often expensive, which means that such tests are often not viable. The objective of this work was therefore to develop an embedded platform for the distributed real-time simulation of dynamic systems. This platform, called the Real-Time Simulator for Dynamic Systems (RTSDS), could be applied in both industrial and academic environments. In industrial applications, the RTSDS could be used to optimize embedded control algorithms. In the academic sphere, it could be used to support research into new embedded solutions for automation and control and could also be used as a tool to assist in undergraduate and postgraduate teaching related to the development of projects concerning on-board control systems.

## 1. Introduction

The development of new embedded algorithms and control techniques for dynamic systems, such as motors, industrial processes, automobiles and aircraft, amongst others, normally requires real-time tests in the devices to be controlled. However, such tests using real systems can be very expensive and time consuming. For example, the refinement of a control algorithm for an automobile injection valve requires tests under real conditions that can last for hours or even days. In other cases, it is necessary to construct small prototypes in order to perform the tests, as in the case of aircraft and ships. Many of these tests under real conditions can be performed using off-line simulations (which are not performed in real time), although such simulations do not fully eliminate real-time testing.

Methods for the development of platforms for real-time simulation have been extensively studied for many years. However, research in this area has recently accelerated following advances in terms of speed and the ease of development associated with new hardware platforms. Methodologies for real-time simulation have included the use of hardware, such as digital signal processors, general-purpose processors and even reconfigurable computational solutions employing field-programmable gate arrays (FPGAs) [1–9]. The benefits of using real-time simulation platforms have been demonstrated in various areas of graduate teaching [10,11]. The combination of real-time simulation with hardware-in-the-loop (HIL) has also been widely investigated in recent years and has brought many benefits in terms of the optimization and prototyping of new control systems [12–17]

In [1], a low-cost real-time simulator (RTS) that can be constructed in the laboratory is proposed for electronic power systems. The system is implemented in a computer with a multi-core processor coupled with data acquisition and signal conditioning boards. The studies described in [2,3] present distributed solutions for RTS in software, where several computers are used in an Ethernet network in order to simulate real-time systems. Signal processing techniques able to reduce the sampling frequency and computational cost associated with RTS are presented in [4], where it is shown that the computational cost can be reduced up to 100-fold, without compromising the precision of the simulation. In [5], a methodology consisting of seven steps is proposed for the validation of real-time simulation models. In [6], a real-time simulation model is proposed for testing AC electrical drives, where the simulation is performed using a Hypersim real-time simulator platform provided by the company, OPAL-RT [18]. Hypersim uses a supercomputer with more than 2000 cores, together with data acquisition boards, and is widely used to simulate large-scale power systems [19]. The use of onboard systems with DSPs is a new approach described in [7], where the dynamic system is implemented using DSPs.

The studies described in [8,9] offer RTS proposals for energy systems employing FPGAs associated with a computer, the main advantage of which is the parallel implementation of the dynamic systems simulated. The benefits of using RTS in the teaching of engineering in graduate and postgraduate courses are described in detail in the works of [10,11], where various commercial RTS tools are presented. Reviews of the advances achieved using real-time simulation schemes with HIL are presented in [12–14], where [13] focuses on applications in energy systems, and [14] focuses on aspects associated with the automotive field. In [15], a real-time simulation system is implemented for testing complex onboard systems with HIL, with the proposed RTS utilizing high-performance hardware with various data acquisition platforms. Finally, modeling and simulation methodologies associated with commercial RTS platforms are presented in [16,17]. The work described in [16] used an RTS provided by the company, dSPACE [20], to simulate wind energy systems, and in [17], an eMEGAsim platform from OPAL-RT [18] was used in an all-electric ship project.

From the cited works, it can be seen that research involving RTS extends from new hardware and software proposals to new modeling techniques aimed at existing commercial systems. It can also be seen that the scope of this area is quite wide and that there is still considerable space for new solutions, especially in the context of simulations employing HIL. The proposal of the present work is therefore to present a new RTS and HIL platform applied to the optimization and testing of control algorithms. This new platform, here called the Real-Time Simulator for Dynamic Systems (RTSDS), possesses a distributed architecture composed of a range of different hardware platforms, enabling it to be constructed in the presence of various resource limitations. As an example, this paper presents a case study in which the RTSDS is composed of a personal computer (Intel DN2800MT Mini-ITX hardware) associated with low-cost and easily-accessible hardware platforms (with embedded software), such as the Arduino and CY8CKIT-001 kits [21–25]. It is important to emphasize that the RTSDS is a solution that does not provide a substitute for high-performance simulation platforms [18,20] that work with sampling times on the order of μs, although it can be used for a range of real systems that work with speeds on the order of ms [26].

The main objective of the Real-Time Simulator for Dynamic Systems (RTSDS) is to provide a support tool for the developers of embedded control systems, focusing on reducing the costs involved in real-time tests. In addition to having industrial applications, the RTSDS could also be used in the academic sphere as a programmable didactic model in various curriculum modules of courses in engineering. Using the RTSDS, the graduate or postgraduate student could test control algorithms in various types of dynamic system, simply and at a cost that is relatively low compared to those of real didactic models. Another point that should be emphasized about the proposal presented here is that the RTSDS can also simulate the functioning of the drivers and sensors associated with the control of dynamic systems, which is an important characteristic from the point of view of the development of control systems projects [27–29].

In order to verify all of the relevant aspects of the RTSDS, this paper presents two case studies in which real-time simulations are made of a level control design and a design for the speed control of a vehicle following a longitudinal route. All of the details concerning the architecture and functioning of the RTSDS are presented here, and all of the source codes can be found in [30], hence enabling the use of this platform by other researchers and students.

## 2. Physical Architecture of the RTSDS

**Figure 1.**
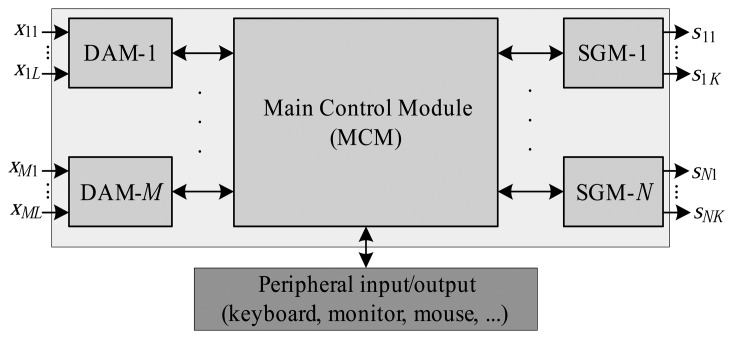
Physical architecture of the Real-Time Simulator for Dynamic Systems (RTSDS) platform.

Figure 1 shows a block diagram of the architecture of the RTSDS. The main control module (MCM) is coupled to a set of *M* data acquisition modules (DAMs) and *N* signal generation modules (SGMs). The function of the MCM is to control, manage and configure the real-time simulation platform, and it is also responsible for the entry and retrieval of data by the user. In principle, the MCM should employ a high-performance processor, which in the present case was the Intel DN2800MT Mini-ITX [31], including an Atom N2800 processor.

The DAMs and SGMs are auxiliary hardware whose function is to receive and generate signals related to the dynamic system to be simulated by the MCM. In practice, the DAMs and SGMs can be implemented by a variety of hardware platforms, such as digital signal processors, microcontrollers and other devices possessing digital and/or analog inputs and outputs. As shown in Figure 1, each *m*-th DAM can include *L* digital and/or analog inputs, and each *n*-th SGM is characterized by *K* digital and/or analog outputs. Each RTSDS must possess at least one DAM and one SGM.

### 2.1. Main Control Module (MCM)

The main control module (implemented with the Intel DN2800MT Mini-ITX hardware) is responsible for configuring and implementing the simulation of the chosen dynamic system. The MCM performs its task by means of an embedded application called the main application (MA), which will be described in the following sections. In addition to the MA, the MCM must possess a set of integrated interfaces and communication protocols in order to achieve communication with the DAMs and SGMs. The RTSDS platform does not demand any specific communication protocol, although the transmission rate *R* (in bps) is an important factor in the functioning of a real-time simulator, especially in the case of fast-response dynamic systems [27].

### 2.2. Data Acquisition Modules (DAMs)

The DAMs are hardware connected to the MCM by means of a data communication protocol transferring bits at a rate of *R*_1_ bps. Each *m*-th DAM can comprise *L* inputs (analog and/or digital) and is responsible for capturing signals from the external environment that will be used by the real-time simulator executed in the MCM. The DAMs utilize an embedded software system, called the application DAM (ADAM). There is a different ADAM for each type of driver and actuator associated with the dynamic system to be simulated. The ADAM is responsible for transforming the *L* external signals connected to the *m*-th DAM into real values and transmitting them to the MCM. The *l*-th input signal of the *m*-th DAM is expressed by *x_ml_*(*t*) and, depending on the driver, may be a variable amplitude and/or frequency analog signal or a variable frequency and/or pulse width digital signal [28].

### 2.3. Signal Generation Modules (SGMs)

These modules are hardware platforms responsible for the generation of signals associated with the outputs of the dynamic system simulated in the MCM. Similar to the DAMs, the SGMs are comprised of *K* outputs (analog and/or digital), communicate with the MCM by means of a data communication protocol at a rate of *R*_2_ bps and employ an embedded software system called the application SGM (ASGM), which converts the simulation data received from the MCM into external analog and/or digital signals. For each type of sensor to be simulated in the dynamic system, there is a different ASGM that converts the output information according to the instrumental characteristics of the sensor in question. The *k*-th output signal of the *n*-th SGM is expressed as *s_nk_*(*t*) and, depending on the type of sensor, may be an analog signal of variable amplitude and/or frequency or a digital signal with variable frequency and/or pulse width [29].

## 3. Logical Architecture of the RTSDS

**Figure 2.**
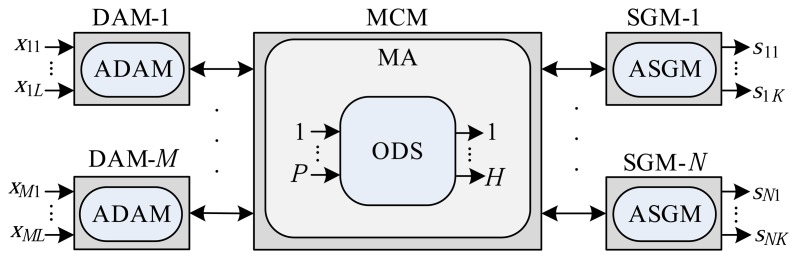
Logical architecture of the RTSDS platform.

Figure 2 illustrates the logical architecture of the RTSDS, where an application located in the MCM, here known as the main application (MA), instantiates a class of software called the object dynamic system (ODS). An ODS represents the dynamic system to be simulated in real time and is composed, amongst others, of eight principal attributes expressed by {*P*, *H*, *f*(*t*), 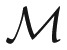, 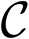, 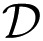, *MethodODE*, *t_a_*}, where:

- *P* represents the number of inputs of the dynamic system.
- *H* represents the number of outputs of the dynamic system.
- *f*(*t*) defines the system of ordinary differential equations (ODEs) of the dynamic system to be simulated in real time.

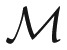
 defines a set of adjustable parameters associated with the dynamic system.

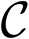
 characterizes the set of embedded codes (the ADAMs) in the DAMs associated with the inputs of the dynamic system.

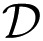
 characterizes the set of embedded codes (the ASGMs) in the SGMs associated with the outputs of the dynamic system.
- *MethodODE* is an attribute that defines which ODE numerical resolution method should be used in the real-time simulation. The RTSDS can implement various resolution methods, including those of Euler, Runge–Kutta, and others [32,33].
- *t_a_* represents the sampling time to be employed in the real-time simulation process.

The RTSDS possesses a database, located in the MCM, composed of various ODSs that can be easily inserted by means of the MA.

The execution of the real-time simulation of an ODS, in the MCM, is achieved using three threads working collaboratively. These are the data acquisition thread (DAT), the simulation thread (ST) and the time control thread (TCT). The function of the DAT is to update all of the *P* inputs of the dynamic system under simulation, reading all of the input buffers associated with the data connections between the MCM and the DAMs. The *P* input variables are shared between the data acquisition and simulation threads. The ST updates the *H* output variables and executes the ODE numerical resolution method (defined by the *MethodODE* variable) in the system of equations, *f*(*t*), in order to update the states of the dynamic system [27,32,33]. The execution of the simulation thread is controlled by the TCT, which uses semaphore logic [34] to regulate the execution time of the ST to a fixed value, *t_s_*, so that the resolution method always occurs at fixed intervals. The use of a fixed interval, *t_s_*, is not essential in numerical resolution methods, but is fundamental for parameterization of the simulation platform, which must have a sampling time, *t_a_*, that is much smaller than the response time, *t_r_*, of the dynamic system to be simulated. In other words, the simulation process must respect the restriction:

(1) $$t_{a}\ll t_{r}$$

**Figure 3.**
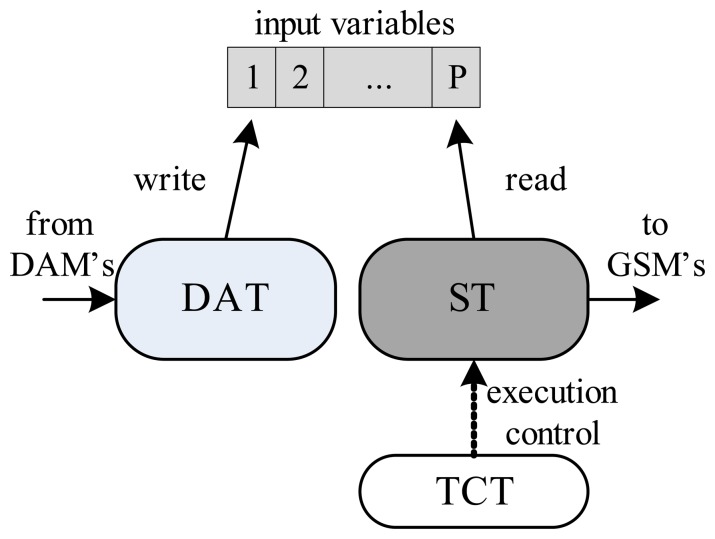
Relationships between the data acquisition thread (DAT), the simulation thread (ST) and the time control thread (TCT).

Figure 3 illustrates the functional relationships between the DAT, ST and TCT.

**Figure 4.**
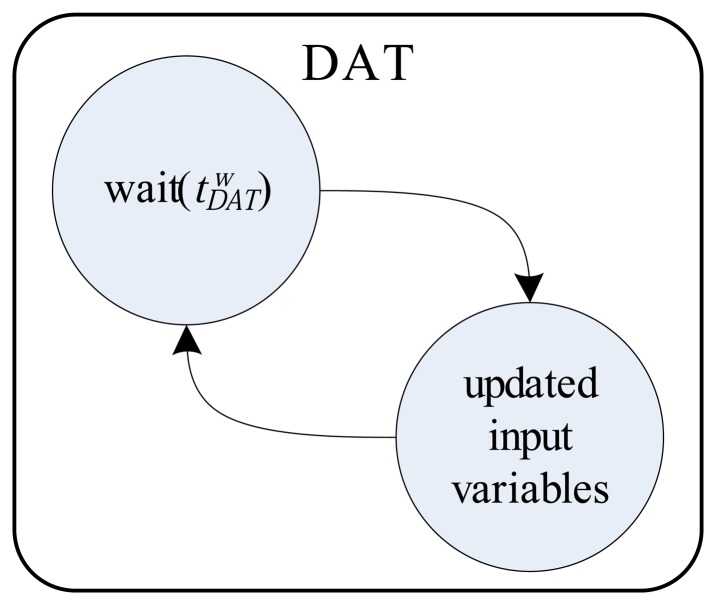
Finite automata associated with DAT.

**Figure 5.**
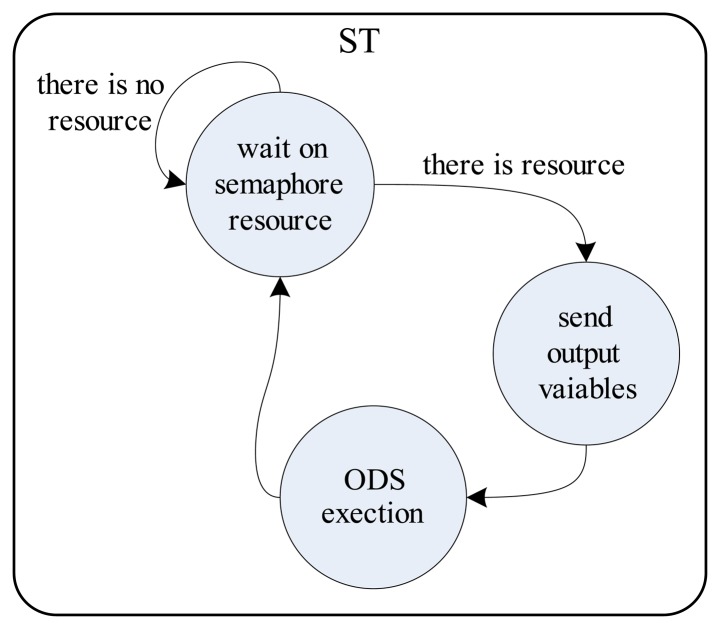
Finite automata associated with ST.

**Figure 6.**
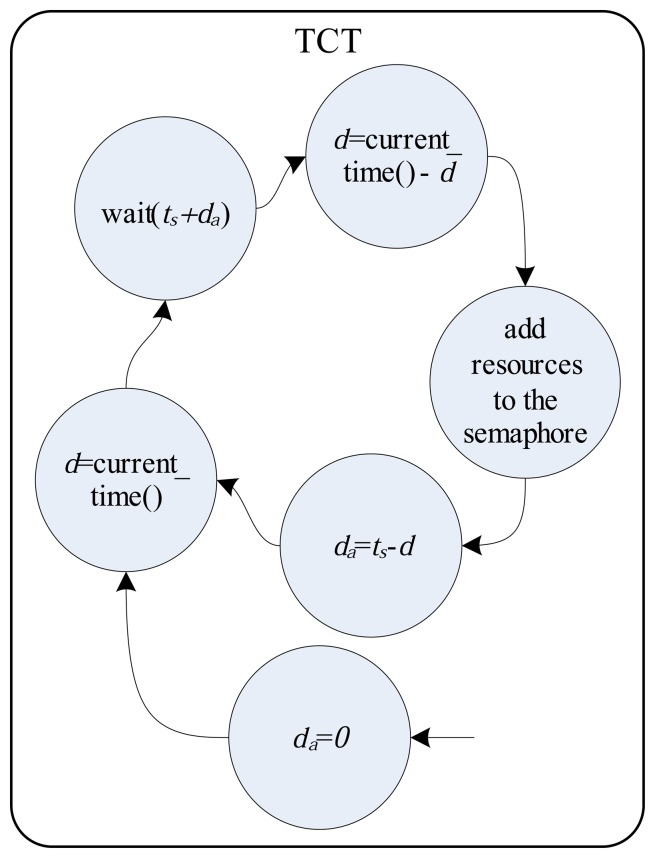
Finite automata associated with TCT.

Figures 4, 5 and 6 detail the finite automata associated with the threads DAT, TS and TCT, respectively. The DAT (Figure 4) scans all communication interfaces buffers with DAMs and updates the *P* input variables of ODS (Figure 3). The DAT is asleep in a predetermined fixed time, $t_{\text{DAT}}^{w}$, to minimize collision problems in the input variables update (write operation), since they are shared with the ST. The finite automata of ST (detailed in Figure 5) wait for the semaphore resource update (by TCT) to send the output variables to SGMs and then run the ODS. Finally, Figure 6 shows, by finite automata, the operation of TCT, which controls the insertion of the semaphore resource. The resource addition is done every, *t_s_*, seconds to ensure the run time of the ODS. The finite automata shown in Figure 6 also detail a system synchronization (using the variables *d_a_* and *d*) that adjusts the run time in *t_s_*.

The codes of the set 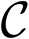 associated with an ODS act as transducers between the external input signal (which can be digital or analog) and one of the *P* inputs of the dynamic system. Each ODS has a one to many relationship with the DAMs and, if necessary, can use all of the *M* DAMs available. The decision of which and how many DAMs will be used by the ODS is made off-line by means of configuration parameters, with the objective of customizing the performance of the system in order to reduce the sampling time, *t_a_*. An important point is that the DAMs can simulate the effect of real drivers and actuators, for example the commercial drivers that work with signals possessing variable frequency and pulse width.

Similar to the DAMs, the role of the set 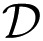 of codes related to the ODS is to act as transducers between the outputs of the dynamic system and the external signals (digital and/or analog) to be generated. Each ODS also has a one to many relationship with the SGMs and can, if necessary, utilize all *N* SGMs associated with the RTSDS. The configuration of which and how many SGMs will be used during the real-time simulation process is configured off-line, as for the DAMs. The SGMs can also simulate the responses of real commercial sensors with variable frequency, amplitude or pulse width.

The simulation of drivers and sensors (using the DAMs and SGMs, respectively) coupled to the dynamic system differentiates the proposed platform, with the simulation process becoming closer to the real case. This benefit is especially important in the development and validation of the embedded control of dynamic systems. Future work will consider the optimization of algorithms for the automatic allocation of DAMs and SGMs to the MCM.

After instantiation of an ODS, the sampling time, *t_a_*, can be expressed by:

(2) $$t_{a}=t_{\text{DAM}}+t_{1}+t_{\text{DAT}}+t_{s}+t_{2}+t_{\text{SGM}}$$

where *t_DAM_* is the fastest processing time associated with all of the DAMs utilized by the MCM, *t*_1_ is the time corresponding to the slowest rate of transfer (in bps) between the MCM and all of the DAMs utilized, *t_DAT_* is the time taken by the DAT to read the information received by all of the DAMs, *t_s_* is the time required for execution of the ST, *t*_2_ is the slowest transfer time (in bps) between the MCM and the SGMs and *t_SGM_* is the fastest processing time associated with all of the SGMs utilized by the MCM. The transmission time, *t*_1_, between a DAM and the MCM can be described by:

(3) $$t_{1}=\frac{b_{1}}{R_{1}}$$

where *b*_1_ is the resolution (in bits) of the value to be transmitted. Similarly, the transmission time between the MCM and the SGM can be expressed by:

(4) $$t_{2}=\frac{b_{2}}{R_{2}}$$

where *b*_2_ is the resolution (in bits) of the value transmitted.

## 4. Description of the Prototype

**Figure 7.**
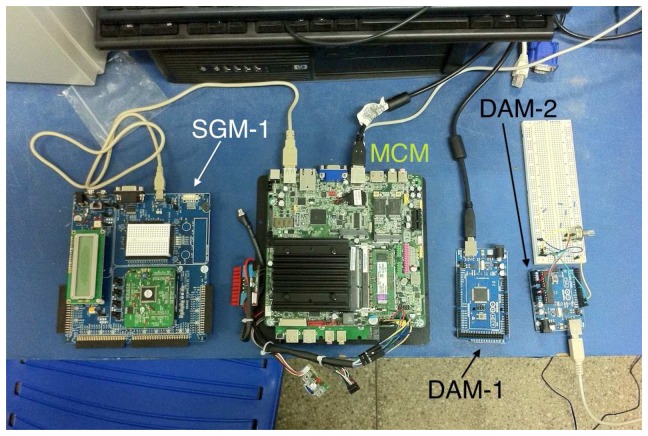
Prototype RTSDS composed of main control module (MCM) data (Intel DN2800MT Mini-ITX), two acquisition modules (DAMs) (Arduino Mega 2560 kit) and a signal generation module (SGM) (PSOC CY8CKIT-001 kit).

Validation of the proposed system involved the development of a prototype (illustrated in Figure 7) with *M* = 2 DAMs (DAM-1 and DAM-2) and *N* = 1 SGM (SGM-1). The MCM was implemented using the Intel DN2800MT Mini-ITX platform with the Linux operating system (distribution: Ubuntu 13.10 [35]), and the DAMs and SGMs were implemented in microcontrollers. DAM-1 utilized an Atmel ATmega 2560 processor [36] with the Arduino Mega 2560 development kit [21]; DAM-2 used an Atmel ATmega 328p processor [22] with the Arduino Uno development kit [23]; and the SGM utilized a Cypress PSOC 3 CY8C38 processor [24] with the PSOC CY8CKIT-001 kit [25]. Communication between the two DAMs and the MCM was accomplished using the USART protocol (Universal Synchronous and Asynchronous Serial Receiver and Transmitter) at a rate of *R*_1_ = 9.6 kbps, and communication between the SGM and the MCM was by means of the USB (Universal Serial Bus) protocol, at a rate of *R*_2_ = 1 Mbps. The main application of the MCM in the prototype was implemented with only one available ODS.

After the development of the prototype, the validation process was continued with the simulation of two dynamic systems whose characteristics are presented in the following subsections. The results associated with the simulations and HIL testing with a PID controller are presented in the next section.

### 4.1. Dynamic System 1: Water in a Tank

**Figure 8.**
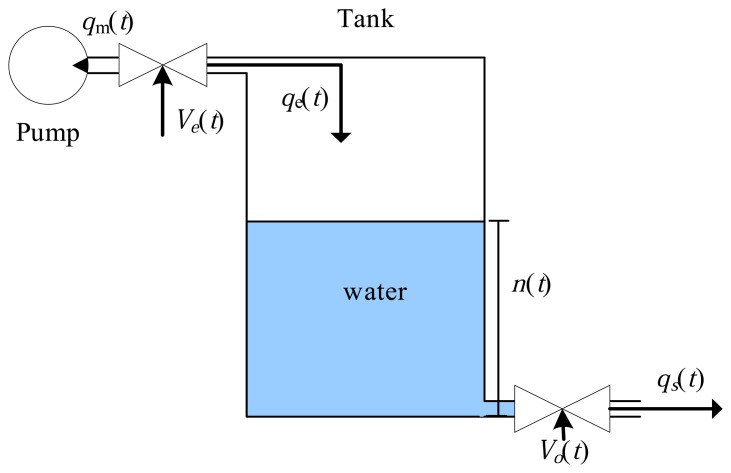
Dynamic system simulated in real time using the prototype.

**Figure 9.**
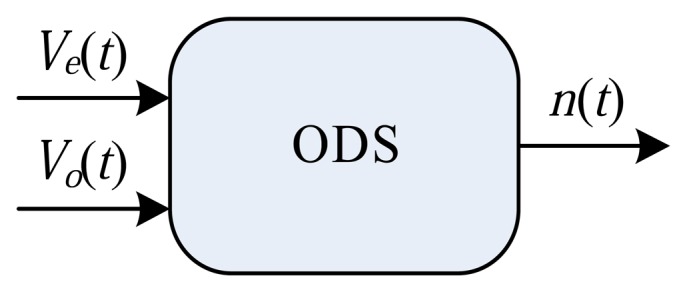
Instantiated object dynamic system (ODS) object composed of two inputs, *P* = 2 (control of the input valve, *V_e_*(*t*), and control of the output valve, *V_o_*(*t*)), and one output, *H* = 1 (level of water in the tank, *n*(*t*)).

This dynamic system is illustrated in Figure 8, and the results are presented in the next section. The real-time simulation involved a tank of water coupled to a pump (see Figure 8). The pump with constant flow, *q_m_*, is coupled to an input valve with continuous control, *V_e_*(*t*), generating a tank input flow of *q_e_*(*t*). The tank output valve also possesses a continuous control, *V_o_*(*t*), and the flow after this valve is given by *q_s_*(*t*). The level of water in the tank is characterized by the *n*(*t*) variable. In this case, the ODS (Figure 9) possesses two inputs, *P* = 2, controlling the input valve, *V_e_*(*t*), and the output valve, *V_o_*(*t*), and one output, *H* = 1, which is the water level in the tank, *n*(*t*). The system of differential equations associated with the ODS can be expressed by:

(5) $$f(t)=\{\begin{array}{l} q_{e}(t)-V_{o}(t)q_{s}(t)=A\frac{dn(t)}{dt}\\ q_{e}(t)=q_{m}V_{e}(t)\\ q_{s}(t)=a\sqrt{2gn(t)} \end{array}$$

where *q_m_*(*t*), *A*, *g* and *a* are the set of adjustable parameters, 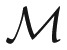, of the ODS, representing the pump flow (cm^2^/s), the transverse section of the tank (cm^2^), the gravitational acceleration (m/s^2^) and the transverse section of the tank output tube (cm^2^), respectively

The dynamic system was simulated in real time, employing the Euler method for ODE resolution (the *MethodODE* variable), which is a simple, but effective method for the dynamic system represented by Equation (5). In this method, Equation (5) can be rewritten as:

(6) $$n(t)=\{\begin{array}{l} z(t)\;\text{for}\;z(t)<n_{\max}\\ n_{\max}\;\text{for}\;z(t)\geq n_{\max} \end{array}$$

where *n_max_* is maximum water level in the tank and:

(7) $$\begin{array}{cc} z(t)=n_{0}+\int_{t_{0}}^{t}h_{tl\;}(s,n(s))ds, & t>t_{0}\;\text{e}\;n_{0}\geq 0 \end{array}$$

where *n*_0_ is the initial condition of the tank level and *h_tl_* (*s, n*(*s*)) is expressed as:

(8) $$h_{tl}(s,n(s))=\frac{1}{A}\left(q_{m}V_{e}(s)-V_{o}(s)a\sqrt{2gn(s)}\right)$$

The Euler method approximates the integral, presented in Equation (7), by the area of a rectangle whose base has length Δ*t* [32,33], in other words,

(9) $$\begin{array}{ll} z(t+\Delta t) & =n_{0}+\int_{t_{0}}^{t}g(s,n(s))ds+\int_{t}^{\Delta t}g(s,n(s))ds\\ & \approx z(t)+g(t,n(t))\times \Delta t \end{array}$$

where Δ*t* is the step of the Euler method [32,33]. For RTSDS, Δ*t* is the run time of the ST, *t_s_* = Δ*t*. Thus, the resolution method implemented in the prototype can be expressed as:

(10) $$n(t+t_{s})=\{\begin{array}{l} \upsilon (t)\;\text{for}\;\upsilon (t)<n_{\max}\\ n_{\max}\;\text{for}\;\upsilon (t)\geq n_{\max} \end{array}$$

where,

(11) $$\upsilon (t)=n(t)+\left(\frac{1}{A}\left(q_{m}V_{e}(t)-V_{o}(t)a\sqrt{2gn(t)}\right)\right)\times t_{s}$$

### 4.2. Dynamic System 2: Longitudinal Vehicle

**Figure 10.**
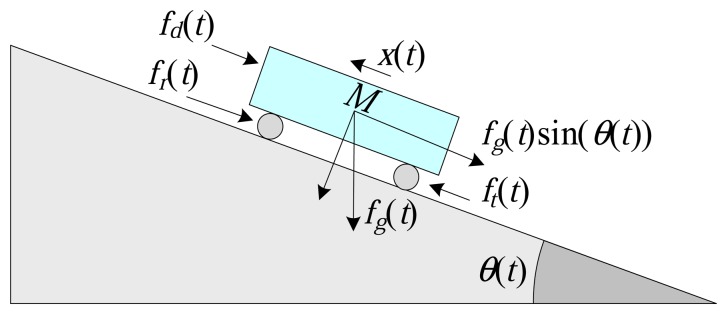
Schematic of the longitudinal vehicle model.

The longitudinal vehicle model [37], illustrated in Figure 10, can be described by the expression:

(12) $$M\frac{dx(t)}{dt}=f_{t}(t)-f_{a}(t)$$

where *M* is the mass of the vehicle (Kg), *x*(*t*) is the linear velocity of the vehicle (m/s), *f_t_*(*t*) is the traction force of the vehicle (N) and *f_a_*(*t*) is the friction force (N). The traction force, *f_t_*(*t*), is expressed as:

(13) $$f_{t}(t)=\frac{\tau_{m}(t)}{r}$$

where *r* is the radius of the wheel of the vehicle (m) and *τ_m_*(*t*) is the torque (Nm) generated by the motor, expressed as:

(14) $$\tau_{m}(t)=V_{e}(t)\tau_{m}^{\max}$$

where $\tau_{m}^{\max}$ is the maximum torque (Nm) and *V_e_*(*t*) is a continuous control signal, similar to Dynamic System 1 (water in a tank).

According to [37], the friction force, *f_a_*(*t*), can be expressed as:

(15) $$f_{a}(t)=f_{d}(t)+f_{r}(t)+f_{g}(t)\sin(\theta (t))$$

where *f_d_*(*t*) is the aerodynamic friction force (N), *f_r_*(*t*) is the rolling resistance force (N), *f_g_*(*t*) is the gravitational force (N) and *θ*(*t*) is the inclination angle of the plane on which the vehicle is located. The variable *θ*(*t*) is expressed as:

(16) $$\theta (t)=V_{o}(t)\theta^{\max}$$

where *θ^max^* is the maximum inclination angle and *V_o_*(*t*) is a continuous control signal, similar to Dynamic System 1 (water in a tank).

The aerodynamic friction can be described by:

(17) $$f_{d}(t)=\frac{1}{2}\rho C_{d}A_{fr}x^{2}(t)$$

where *ρ* is the density of air, *C_d_* is the aerodynamic drag coefficient and *A_fr_* is the frontal area of the vehicle (m^2^). The rolling resistance force can be described by:

(18) $$f_{r}(t)=Mg(C_{0}+C_{1}x^{2}(t))$$

where *C*_0_ and *C*_1_ are the rolling coefficients and *g* is the acceleration due to gravity (m/s^2^). Finally, the gravitational force is given by:

(19) $$f_{g}(t)=Mg$$

The dynamic system was simulated in real time, employing the Euler method for ODE resolution (the *MethodODE* variable). In this method, Equation (12) can be rewritten as:

(20) $$x(t)=\{\begin{array}{l} u(t)\;\text{for}\;u(t)<x_{\max}\\ x_{\max}\;\text{for}\;u(t)\geq x_{\max} \end{array}$$

where *x_max_* is maximum vehicle speed and:

(21) $$\begin{array}{cc} u(t)=x_{0}+\int_{t_{0}}^{t}h_{\upsilon s}(s\;,x(s))\;ds, & t>t_{0}\;\text{e}\;x_{0}\geq 0 \end{array}$$

where *x*_0_ is the initial condition of the vehicle speed and *h_υs_* (*s*, *x*(*s*)) is expressed as:

(22) $$h_{\upsilon s}(s,x(s))=\frac{1}{M}\left(V_{e}(s)\frac{\tau_{m}^{\max}}{r}-\frac{1}{2}\rho C_{d}A_{fr}x^{2}(s)-Mg(C_{0}+C_{1}x^{2}(s))-Mg\sin(V_{o}(s)\theta^{\max})\right)$$

The Euler method approximates the integral, presented in Equation (21), by the area of a rectangle whose base has length Δ*t* [32,33], in other words,

(23) $$\begin{array}{ll} u(t+\Delta t) & =x_{0}+\int_{t_{0}}^{t}h_{\upsilon s}(s,x(s))\;ds+\int_{t}^{\Delta t}h_{\upsilon s}(s,x(s))\;ds\\ & \approx u(t)+h_{\upsilon s}(t,x(t))\times \Delta t \end{array}$$

where Δ*t* is the step of the Euler method [32,33]. For RTSDS, Δ*t* is the run time of the ST, *t_s_* = Δ*t*. Thus, the resolution method implemented in the prototype can be expressed as:

(24) $$x(t+t_{s})=\{\begin{array}{l} \upsilon (t)\;\text{for}\;c(t)<x_{\max}\\ x_{\max}\;\text{for}\;c(t)\geq x_{\max} \end{array}$$

where,

(25) $$c(t)=x(t)+\left(\frac{1}{M}\left(V_{e}(t)\frac{\tau_{m}^{\max}}{r}-\frac{1}{2}\rho C_{d}A_{fr}x^{2}(t)-Mg(C_{0}+C_{1}x^{2}(t))-Mg\sin(V_{o}(t)\theta^{\max})\right)\right)\times t_{s}$$

**Figure 11.**
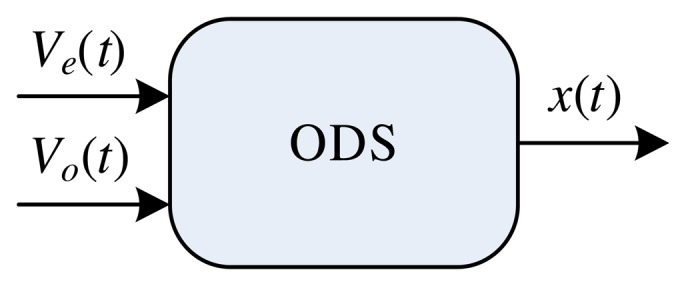
Instantiated ODS object composed of two inputs, *P* = 2 (control of the traction force, *V_e_*(*t*), and control of the inclination angle, *V_o_*(*t*)), and one output, *H* = 1 (speed of the vehicle in the longitudinal direction, *x*(*t*)).

In this case, the ODS (Figure 11) possesses two inputs, *P* = 2, controlling the traction force, *V_e_*(*t*), as well as the inclination angle, *V_o_*(*t*), and one output, *H* = 1, which is the speed of the vehicle in the longitudinal direction, *x*(*t*).

### 4.3. General Parameters

The control signal, *V_e_*(*t*), can have values of between zero and one in the ODS. In order to represent a more real situation, the external signal, *x*_11_(*t*), associated with *V_e_*(*t*) consists of a digital signal with a fixed frequency and a pulse width that can be varied between 0% and 100% (pulse-width modulation, PWM) [28]. Hence, the DAM-1, using an embedded ADAM (set 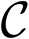 of codes) in the ATmega 2560, can recognize the PWM pulse width *x*_11_(*t*) in real time and convert it to a value of between zero and one, which is transmitted to the MCM via the USART protocol at a rate of *R*_1_ = 9.6 kbps. The resolution of the value associated with *V_e_*(*t*) in conversion of the PWM signal was eight bits (*b*_1_ = 8), in order to simplify the transmission process, resulting in a transmission time of 0.83 ms. The embedded application in the DAM-1 had an execution time of around 110 ms, due to the frequency of 100 Hz utilized in the PWM.

In the case of the control signal, *V_o_*(*t*), the ODS also operates with values of between zero and one. However, in order to differentiate this case from the preceding case, the external signal, *x*_21_(*t*), associated with *V_o_*(*t*), has an amplitude that is variable between zero and 2.5 volts. In this way, the DAM-2, using an embedded ADAM (set 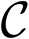 of codes) in another ATmega 2560, recognizes the amplitude of the signal *x*_21_(*t*) in real time and converts it to a value of between zero and one for transmission to the MCM by means of the USART protocol at a rate of *R*_1_ = 9.6 kbps. The resolution of the value associated with *V_o_*(*t*) in the analog/digital conversion was eight bits (*b*_1_ = 8), resulting in a transmission time of 0.83 ms. The embedded application in the DAM-2 had an execution time of around 20 ms.

Finally, the output of the simulated dynamic system, *n*(*t*) (level in the tank) or *x*(*t*) (vehicle speed), was associated with an analog signal, *s*_11_(*t*), in SGM-1. In this case, the embedded ASGM (set 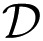 of codes) in the SGM-1 simulates an analog sensor of a type widely used in industry, with output values of between zero and one volt. A resolution of eight bits was used for the value of the level output, *n*(*t*), with a transmission rate of 1 Mbps (via USB protocol) between the MCM and SGM-1, resulting in a transmission time of around 8 μs. The resolution of the digital/analog converter in SGM-1 was eight bits, and the execution time of the ASGM was approximately 1 ms.

Table 1 summarizes the times estimated for the DAMs and SGMs, based on the variables presented in Equation (2). From these values and the response time of the dynamic system, *t_r_*, it is possible to estimate the value of *t_s_* and calculate the sampling time of the system, *t_a_*.

## 5. Experimental Results

**Figure 12.**
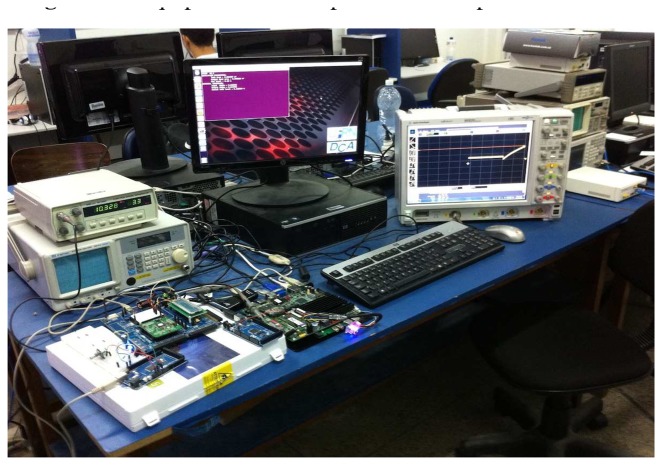
Equipment used to perform the experimental tests.

The prototype developed was validated experimentally by varying the set of parameters, 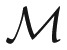 = {*q_m_*(*t*), *A*, *g*, *a*}, of the dynamic system characterized by Equation (5). The results of these tests were compared with those obtained using the MATLAB/Simulink platform [38] in simulations that were not performed in real time. In the case of Dynamic System 1, the values employed for the parameters of the set 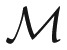 were based on a didactic kit of coupled tanks, supplied by Quanser [39], which is used in disciplines involving control and automation systems. For Dynamic System 2, the values are based on previous field tests of electric vehicles [40,41]. Figure 12 shows the equipment used to perform the experimental tests.

### 5.1. Results of Dynamic System 1: Water in a Tank

In all of the experiments with Dynamic System 1, the pump flow was held constant at *q_m_*(*t*) = 69 cm^3^/s; the acceleration due to gravity was *g* = 9.8 m/s^2^; the maximum level of the tank was *n_max_* = 30 cm; and the transverse section of the output tube was *a* = 0.1781 cm^2^, generating a maximum outlet flow, *q_s_*(*t*), of 17.81 cm^3^/s. The tests used values of *A* = 15.518 cm^2^ and *A* = 62.072 cm^2^ for the area of the transverse section of the tank, so that in the worst case, the response time of the dynamic system, *t_r_*, was in the region of *t_r_* ≈ 100 s. Based on the value of *t_r_* and the values presented above (summarized in Table 1), use of Equation (2) gives an estimated value of *t_s_* of 100 ms, while the calculated value of *t_a_* was 212 ms, taking into account the restriction presented in Equation (1).

**Figure 13.**
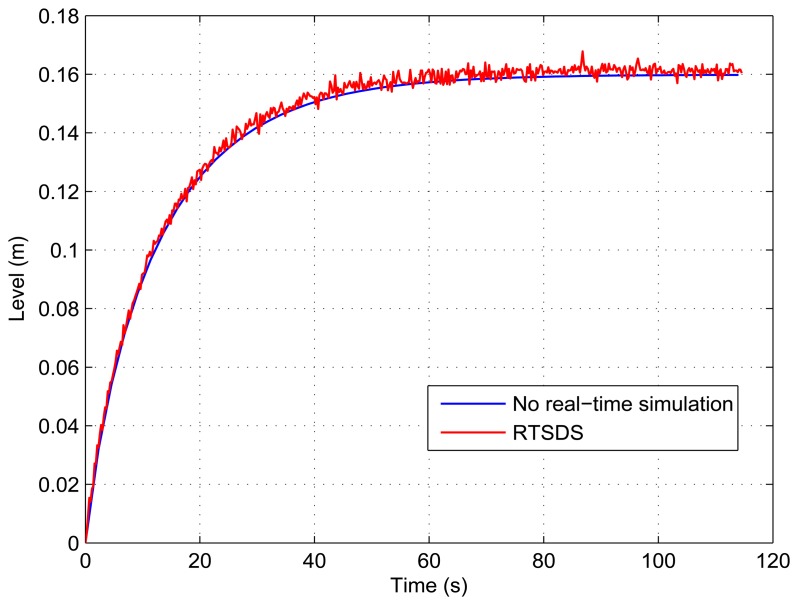
Results obtained for the RTSDS platform applied to the dynamic system characterized by Equation (5), with *A* = 15.518 cm^2^ and *q_e_*(*t*) = 0.457.

**Figure 14.**
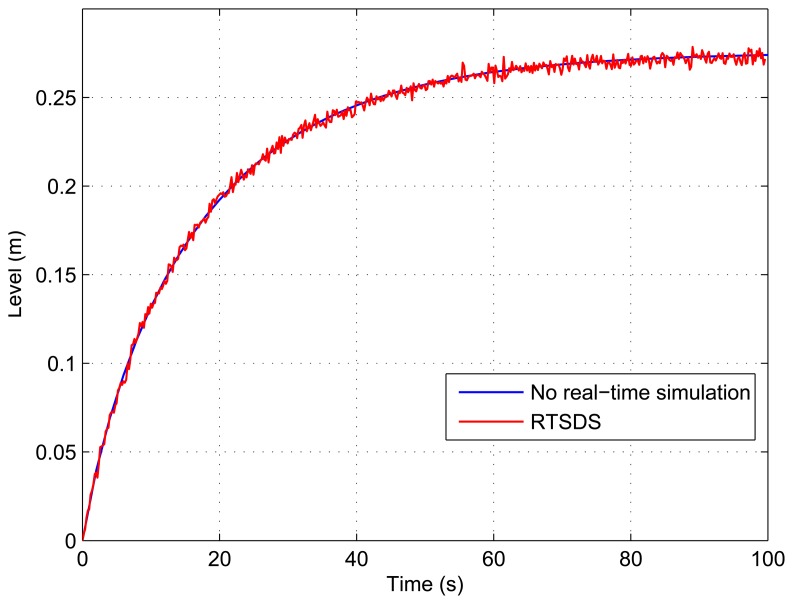
Results obtained for the RTSDS platform applied to the dynamic system characterized by Equation (5), with *A* = 15.518 cm^2^ and *q_e_*(*t*) = 0.6.

**Figure 15.**
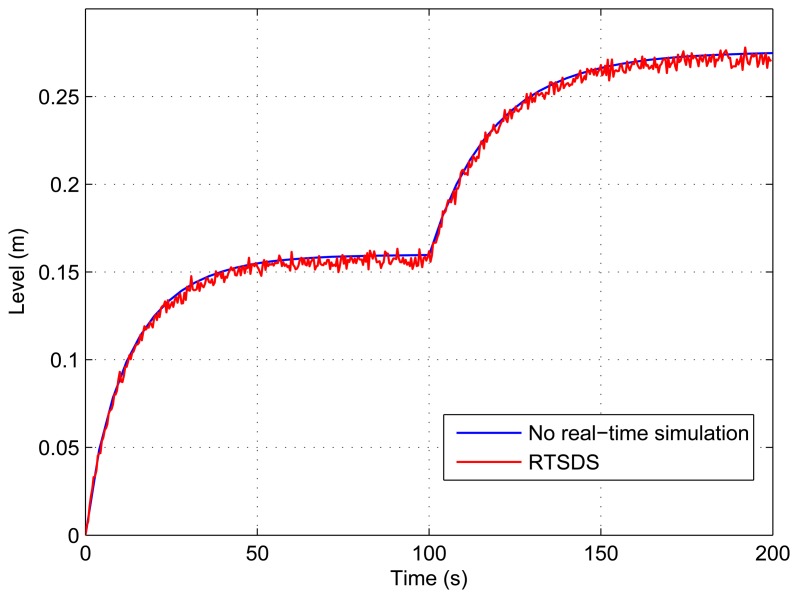
Results obtained for the RTSDS platform applied to the dynamic system characterized by Equation (5), with *A* = 15.518 cm^2^ and *q_e_*(*t*) = 0.457 initially and then *q_e_*(*t*) = 0.6 after 100 s.

**Figure 16.**
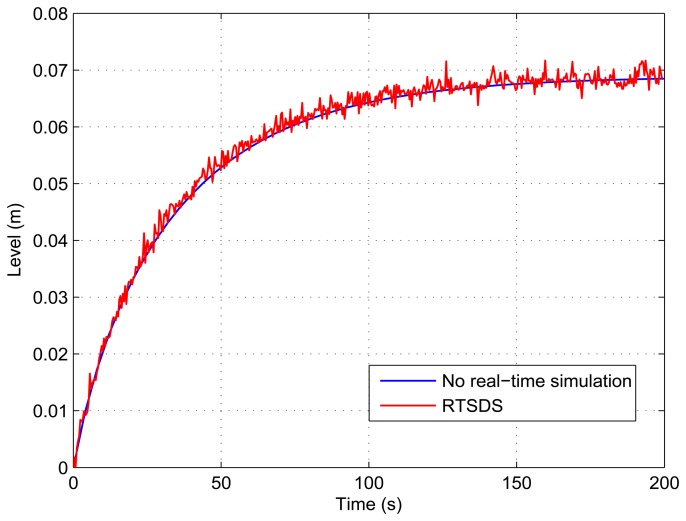
Results obtained for the RTSDS platform applied to the dynamic system characterized by Equation (5), with *A* = 62.072 cm^2^ and *q_e_*(*t*) = 0.3.

**Figure 17.**
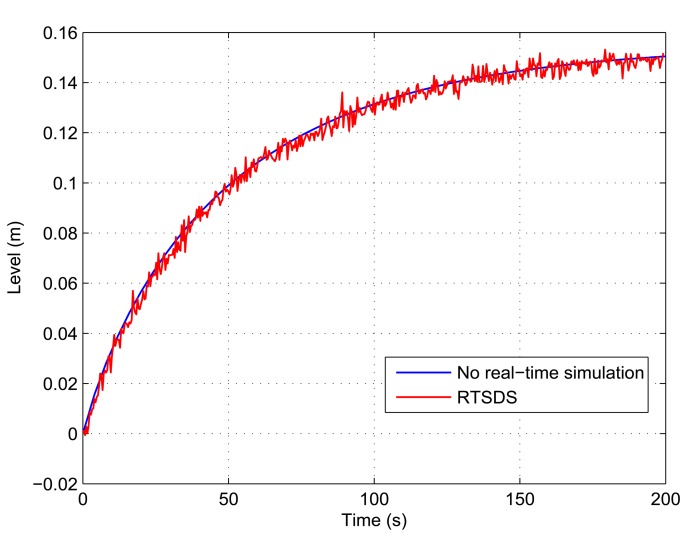
Results obtained for the RTSDS platform applied to the dynamic system characterized by Equation (5), with *A* = 62.072 cm^2^ and *q_e_*(*t*) = 0.45.

Figures 13, 14, 15, 16 and 17 show the results obtained for the RTSDS platform used to execute the prototype described above, together with the non-real-time results obtained using MATLAB/Simulink. In all cases (with Dynamic System 1), it can be seen that the RTSDS platform provided satisfactory results, with errors that were very small in relation to the reference method. In all of the tests with Dynamic System 1 (water in a tank) (Figures 13, 14, 15, 16 and 17), the outlet flow volume was fixed at the maximum value, *V_o_*(*t*) = 1, and the inlet flow volume, *V_e_*(*t*), was adjusted between zero and one, varying the inlet flow rate, *q_e_*(*t*), relative to the constant flow produced by the pump, *q_m_*(*t*) (see Equation (5)).

The results for a tank with *A* = 15.518 cm^2^ are shown in Figures 13 and 14 for *q_e_*(*t*) = 0.457 and *q_e_*(*t*) = 0.6, respectively. Figure 15 presents the results for a programmed sequence of perturbations, with DAM-1 being programmed to begin with *q_e_*(*t*) = 0.457 and then after 100 s to change to *q_e_*(*t*) = 0.6. Figures 16 and 17 present the results for a tank with *A* = 62.072 cm^2^ and *q_e_*(*t*) = 0.3 and *q_e_*(*t*) = 0.45, respectively.

**Figure 18.**
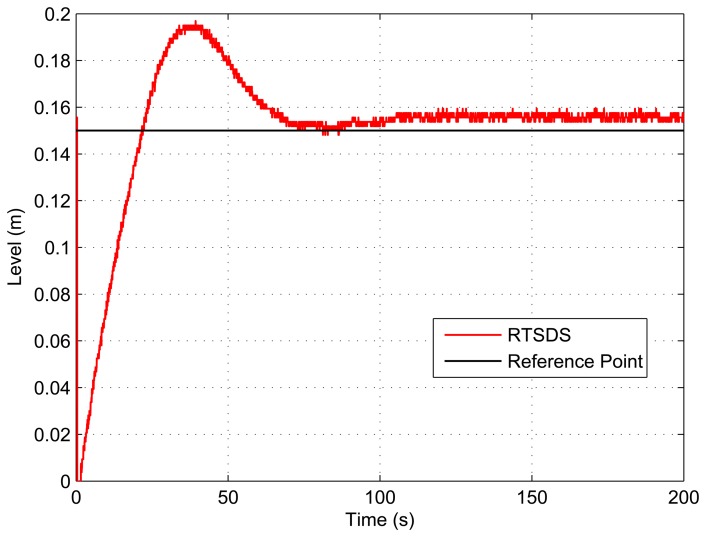
Results obtained for the RTSDS platform utilizing an external PID controller (*k_p_* = 2, *k_i_* = 0.2 e *k_d_* = 0.001) onboard an ATmega 2560 microcontroller.

**Figure 19.**
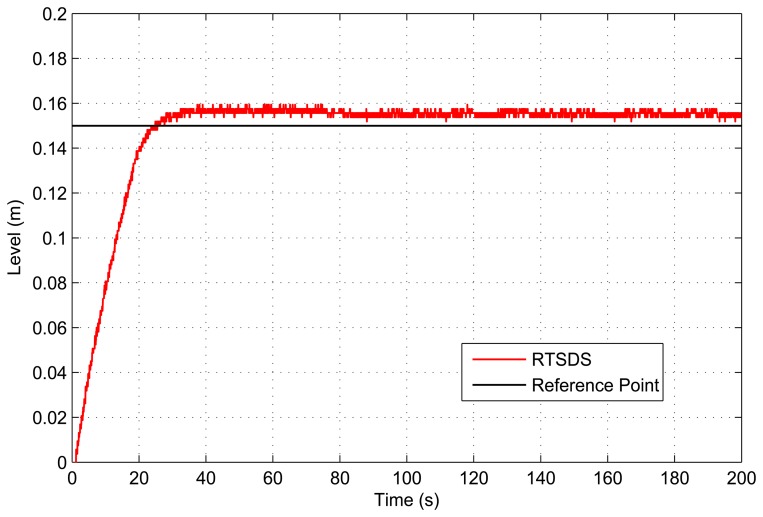
Results obtained for the RTSDS platform utilizing an external PID controller (*k_p_* = 5, *k_i_* = 0.1 e *k_d_* = 0.03) onboard an ATmega 2560 microcontroller.

**Figure 20.**
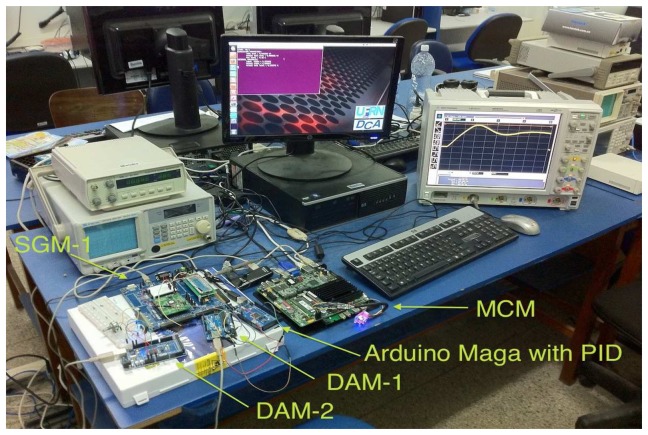
Equipment used in testing with the PID.

Figures 18 and 19 present the results obtained for an embedded PID controller [27] in an ATmega 2560 microcontroller (utilizing the Arduino Mega 2560 kit) external to the RTSDS platform. The system was controlled to a reference value (set point) of 15 cm, and the results show the response of the system in terms of the value associated with the level, *n*(*t*). This experiment clearly demonstrated the benefits derived from using the RTSDS in the development of embedded control algorithms, where the performance and viability of new algorithms could be tested in real time, without any need to acquire expensive physical equipment. Figure 20 shows the equipment used in testing with the PID.

### 5.2. Results of Dynamic System 2: Longitudinal Vehicle

In all of the experiments with Dynamic System 2, the maximum torque was held constant at $\tau_{m}^{\max}=2000\text{Nm}$; the acceleration due to gravity was *g* = 9.8 m/s^2^; the maximum speed of the vehicle was *x_max_* = 200 m/s; the mass of the vehicle was *M* = 450 Kg; the density of air was *C_d_* = 1.18; the aerodynamic drag coefficient was *C_d_* = 0.51; the frontal area of the vehicle was *A_fr_* = 2.4 m^2^; the radius of the wheel was *r* = 0.26 m; the maximum inclination angle was *θ^max^* = 60 degrees ; and the rolling coefficients were *C*_0_ = 0.015 and *C*_1_ = 0.

The *t_r_* was in the region of *t_r_* ≈ 100 s. Based on the value of *t_r_* and the values presented above (summarized in Table 1), use of Equation (2) gives an estimated value of *t_s_* of 100 ms, while the calculated value of *t_a_* was 212 ms, taking into account the restriction presented in Equation (1).

**Figure 21.**
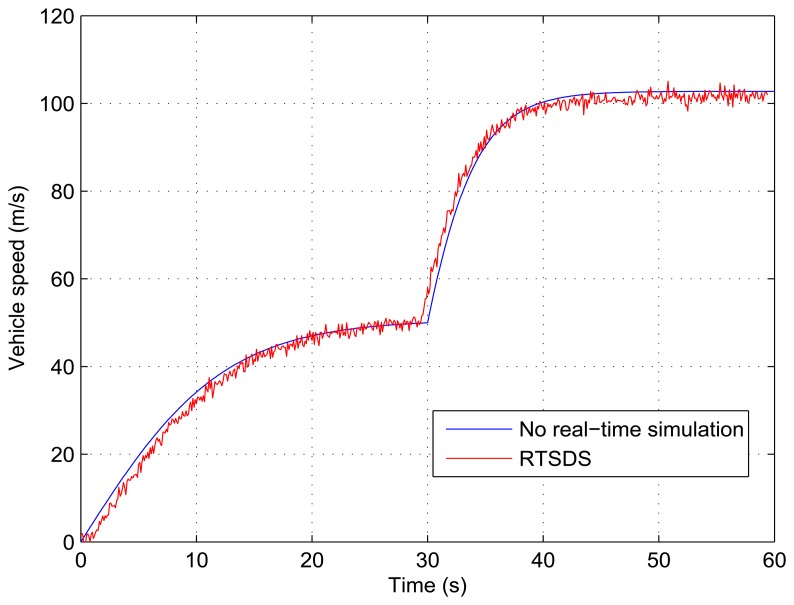
Results obtained for the RTSDS platform applied to the dynamic system characterized by Equation (12), with *θ*(*t*) = 0 degree and *τ_m_*(*t*) = 500 Nm initially and, then, *τ_m_*(*t*) = 2000 Nm after 30 s.

**Figure 22.**
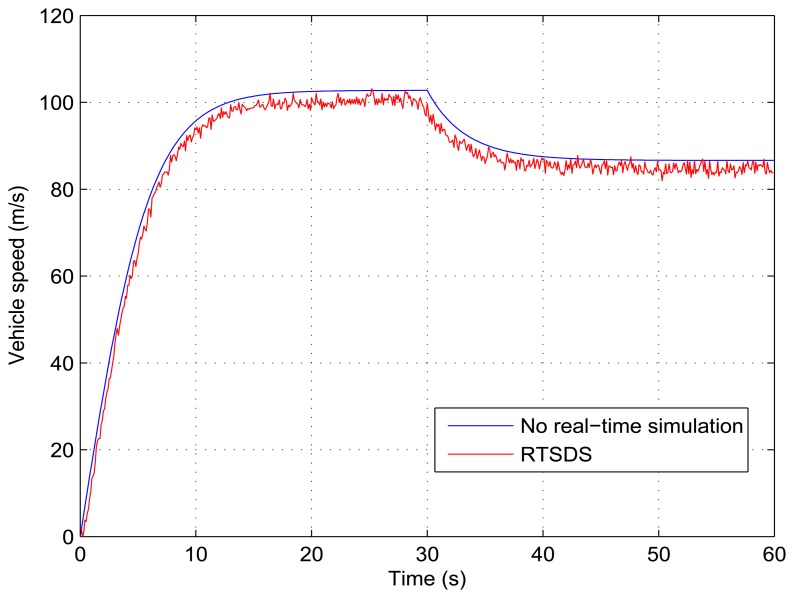
Results obtained for the RTSDS platform applied to the dynamic system characterized by Equation (12), with *τ_m_*(*t*) = 2000 Nm and *θ*(*t*) = 0 degree initially and, then, *θ*(*t*) = 30 degree after 30 s.

Figures 21 and 22 show the results obtained for the RTSDS platform used to execute the prototype described above, together with the non-real-time results obtained using MATLAB/Simulink. Figure 21 presents the results for a programmed sequence of perturbations, with DAM-1 being programmed to begin with *τ_m_*(*t*) = 500 Nm (*V_e_*(*t*) = 0.25) and, then, after 2000 Nm (*V_e_*(*t*) = 1) with *θ*(*t*) = 0 degree (*V_o_*(*t*) = 0). Figure 22 presents the results for a programmed sequence of perturbations, with DAM-2 being programmed to begin with *θ*(*t*) = 0 degree (*V_o_*(*t*) = 0) and, then, after 30 degree (*V_o_*(*t*) = 0.5) with *τ_m_*(*t*) = 2000 Nm (*V_e_*(*t*) = 1). In all cases with Dynamic System 2 (Longitudinal Vehicle), it can be seen that the RTSDS platform provided satisfactory results, with errors that were very small in relation to the reference method.

**Figure 23.**
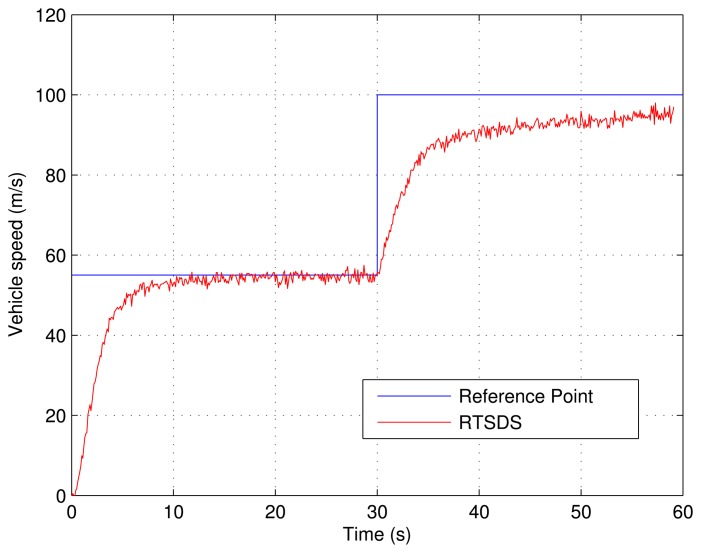
Results obtained for the RTSDS platform utilizing an external PID controller (*k_p_* = *3*, *k_i_* = 0.2 e *k_d_* = 0.002) onboard an ATmega 2560 microcontroller.

Figure 23 presents the results obtained for an embedded PID controller [27] in an ATmega 2560 microcontroller (utilizing the Arduino Mega 2560 kit) external to the RTSDS platform. The system was controlled to begin with a reference value (set point) of 55 m/s and, then, after 100 m/s.

## 6. Conclusions

This work presents a proposal for an embedded solution for the real-time simulation of dynamic systems, called the RTSDS. This platform is composed of a central hardware module (the MCM) and a set of ancillary hardware modules (the DAMs and SGMs), so that the platform is modular and distributed. Experimental trials were performed using a prototype developed using an Intel DN2800MT Mini-ITX kit as the MCM, Arduino Mega 2560 kits as the DAMs and a PSOC CY8CKIT-001 kit as the SGM. The results obtained showed that the RTSDS platform provided good performance, with errors that were very small, compared to simulations that were not performed in real time. The RTSDS could be used to optimize and synchronize embedded control algorithms used in industry and also as a learning tool to support graduate and postgraduate teaching.

## Figures and Tables

**Table 1.** Estimated times for the DAMs and SGMs, based on the variables presented in Equation (2).

**Times**	DAM-1	DAM-2	SGM-1	**MCM**	**Selected**
*t_DAM_*	110 ms	20 ms	-	-	110 ms
*t*_1_	0.83 ms	0.83 ms	-	-	0.83 ms
*t_SGM_*	-	-	1 ms	-	1 ms
*t*_2_	-	-	8 μs	-	8 μs
$t_{\text{DAT}}\left(t_{\text{DAT}}^{w}=100\mu \text{s}\right)$	-	-	-	200 μs	200 μs
